# Oral health knowledge, attitudes, behaviours and status among international post-secondary students: a scoping review

**DOI:** 10.3389/froh.2025.1555165

**Published:** 2025-03-21

**Authors:** Hassan W. Yassin, Shahzaib Fida, Khrisha Alphonsus, Jessica Lieffers, Amrinderbir Singh

**Affiliations:** ^1^ College of Dentistry, University of Saskatchewan, Saskatoon, SK, Canada; ^2^ School of Public Health, University of Saskatchewan, Saskatoon, SK, Canada; ^3^ College of Pharmacy and Nutrition, University of Saskatchewan, Saskatoon, SK, Canada

**Keywords:** oral health, international student, knowledge attitudes and behaviors (KABs), oral health and nutrition, vulnerable population, determinants of oral health, nutrition, access to oral health care

## Abstract

**Objective:**

This scoping review aims to review and synthesize existing literature on oral health knowledge, attitudes, behaviours, barriers, and status among international post-secondary students.

**Methods:**

Using the Arksey and O'Malley framework, MEDLINE, Embase, Dentistry & Oral Sciences Source, CINAHL, Web of Science, and Scopus databases were searched in June 2024 for selected oral health and international student keywords. Manual searches of reference lists and citations were also conducted. Original research studies in English language were included, with no geographical or date limitations. Using Rayyan, duplicates were removed, and then two authors independently screened available literature according to eligibility criteria; inconsistencies or disagreements were resolved through a third author.

**Results:**

The search yielded 984 articles. After removal of duplicates and those inconsistent with our inclusion criteria, 14 articles remained. In total, 13/14 articles used a cross-sectional design implementing surveys or interviews; only 4 articles presented objective clinical measures (e.g., DMFT, objective periodontal measures). Some included articles provided information about dietary habits relevant to oral health; however, information captured was limited. Overall, compared to the domestic students, international students were reported to have poorer oral health status; more gaps in their knowledge, attitudes and behaviours regarding oral health; and were also less likely to obtain routine oral health care.

**Conclusion:**

International students may face significant challenges in managing and optimizing their oral health vs. domestic students due to various factors (e.g., acculturation stress, finances, diet, academic stress etc.). Post-secondary institutions may want to consider focusing on supporting and empowering international students to access oral health care on a regular basis through targeted interventions. To design impactful interventions, future community engaged research is needed to better understand the perspectives of international students regarding their oral health status, knowledge, attitudes, behaviours, needs, and aspirations.

## Introduction

Oral health is essential for overall well-being and substantially impacts an individual's quality of life. According to the FDI World Dental Federation, oral health involves a wide range of functions and expressions, including the abilities to speak, smile, taste, and chew, as well as to show emotions through facial expressions without experiencing pain, discomfort, or diseases affecting the head, face and oral cavity (1). Despite its importance for overall health, oral health frequently receives less attention compared to other aspects of health, resulting in a widespread prevalence of oral diseases worldwide.

Post-secondary students face unique challenges in maintaining good oral health. These individuals often experience demanding academic schedules, newfound independence, and changes in financial status, which can lead to difficulties maintaining oral hygiene practices and care (2). Moreover, this life stage can be accompanied by poor diet quality, stress, and the use of substances such as tobacco and alcohol which further compromise oral health (3). These factors collectively create a challenging environment for students to prioritize and maintain good oral health behaviours.

Within this demographic, international students are a population who face additional challenges (4). These challenges include sociocultural adjustment issues, such as homesickness, loneliness, cultural differences, academic difficulties, language barriers, and adapting to new educational systems (4). Skromanis et al. noted that international students were at a heightened risk for several adverse health outcomes including psychological distress, substance use, and problem gambling (5). These adverse health outcomes are fueled by cultural and academic adjustments and a lower likelihood of seeking help for health issues compared to their domestic counterparts. The significance of these multifaced challenges is magnified when considering the global scale of international education, with over 6 million students studying outside their home countries in 2021 (6).

This scoping review aims to explore existing literature on oral health knowledge, attitudes, behaviours, barriers, and status among international post-secondary students. Despite the vital role of oral health in overall well-being and the large number of international students worldwide, reviews focusing on oral health in this population remain scarce. By synthesizing current evidence, the review seeks to highlight key findings, identify research gaps, and provide recommendations for future research and practice. This review will contribute to a better understanding of the portrait of oral health in international students. These findings will also support the development of targeted interventions to improve oral health behaviours, outcomes, and access to care related challenges in this population.

## Materials and methods

Our scoping review followed the framework developed by Arksey and O'Malley and later updated by Levac et al. (7, 8) The Preferred Reporting Items for Systematic Reviews and Meta-Analysis Extension for Scoping Reviews (PRISMA-ScR) also guided this review (9).

### Stage 1: Defining the research question

Our research question was: *What is known about the knowledge, attitudes, behaviours, and status regarding oral health of international students at post-secondary institutions?* A scoping review was chosen as we were interested in understanding the range and depth of literature available on this topic and creating a summary to provide insights for researchers, practitioners and stakeholders interested in this area. We also sought to identify knowledge gaps to help guide further research activities surrounding this topic. Our preliminary search indicated that there is a paucity of research in this area, underscoring the need for further exploration.

### Stage 2: Identification of relevant studies

Databases searched in June 2024 included MEDLINE, Embase, Dentistry & Oral Sciences Source, CINAHL, Web of Science, and Scopus. We used a combination of controlled language (e.g., MeSH terms, Emtree) and keywords as appropriate. We searched two concepts: (1) oral health (e.g., oral health, periodontitis, oral hygiene, dentistry, tooth, teeth, gingivitis) and (2) international students (e.g., international student, overseas student, foreign-born student, exchange student). The search concepts were combined using the “AND” Boolean operator. A health sciences librarian provided feedback on search strategies. The search terms (Supplementary Material 1) were initially established using MEDLINE and modified accordingly to search other databases. In addition to searching databases, we reviewed the reference lists of all included articles for any additional literature for possible inclusion. We also checked citations of all included articles in Google Scholar for any additional studies for possible inclusion.

### Stage 3: Article selection

We included original research studies that investigated post-secondary international students' oral health knowledge, attitudes, practices and/or oral health status; and were published in English. We had no restrictions on geographical location and publication date. Studies were excluded that (i) solely focused on student populations with advanced oral health knowledge (e.g., international dental and medical students); (ii) considered refugee students as international students; (iii) were published as conference abstracts, posters, or case studies; or (iv) reported combined data on international and domestic students (i.e., did not have exclusive international student data). Duplicate articles were removed using the functions available in Rayyan (Rayyan, Qatar). After removal of duplicates, two authors (HY, SF) independently screened all available literature in Rayyan using the listed inclusion and exclusion criteria. Any inconsistencies or disagreements regarding article inclusion were resolved through discussion with a third author (JL) providing a final decision if needed.

### Stage 4: Charting the data

Data extraction was performed by two reviewers (HY, SF). Key details from each study were extracted including author(s), year of publication, study objectives, study design, country, sampling method, sample size, participant characteristics (e.g., age, gender, country of origin, level of education), data collection tools, results, conclusions, and recommendations or identified research gaps. The information was charted using Google Sheets.

### Stage 5: Data synthesis

Google Sheets was used to organize the extracted article information into themes and connect article information to describe the study characteristics. These analyses helped to identify gaps in the existing literature.

## Results

**Figure 1:**
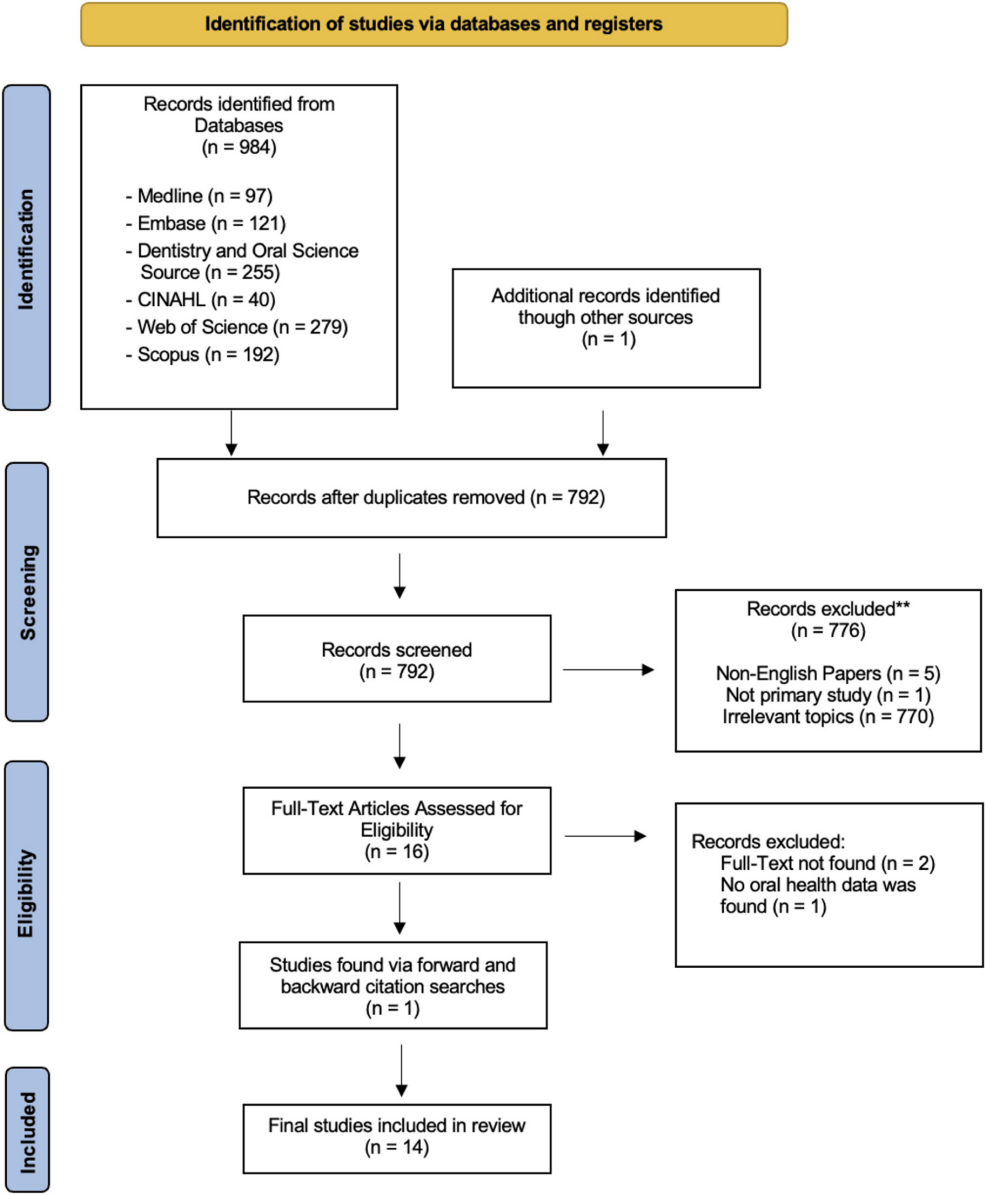
PRISMA flow diagram.

From the database searches, 984 articles were obtained (MEDLINE: *n* = 97; Embase: *n* = 121, Dentistry & Oral Sciences Source: *n* = 255, CINAHL: *n* = 40, Web of Science: *n* = 279, Scopus: *n* = 192). After duplicate screening, 792 articles remained. Following the title and abstract screening, 16 full-text articles were assessed for eligibility. After full-text screening, 14 articles met the eligibility criteria and were included (Figure 1).

### Study characteristics

The 14 studies were conducted in diverse locations (Table 1). Three studies were carried out in each of the following countries: Brazil (10–12), United States (13–15), and Korea (16–18). Two studies were conducted in Japan (19, 20), and one study occurred in each of the following countries: Thailand (21), Georgia (22), and Malaysia (23). Overall, one study was published before 2010 (13), six studies were published between 2010 and 2019 (14–19), and seven studies were published in 2020 or later (10–12, 20–23).

**Table 1:** Article location and methodology.

Author(s), Date	Location	Methodology
(Susanti et al., 2023) (21)	Thailand	Cross-sectional study conducted using Google Forms. Online surveys were distributed to post-secondary students. Questionnaires were evaluated for validity using IOC (Item-Objective Congruence Index) by 3 experts in oral healthcare.
(Tsitaishvili & Jikia, 2023) (22)	Georgia	Randomized research study with self-assessment surveys. Post-secondary students received basic periodontal examinations conducted by a dentist to observe oral hygiene status. Self-administered questionnaires were distributed as well.
(Abe et al., 2023) (20)	Japan	Retrospective review of post-secondary students’ clinical data. Periodontal health status was assessed using bleeding on probing, calculus deposition, and probing pocket depth.
(Cruz et al., 2022) (11)	Brazil	Transversal and analytic study with a quantitative approach that utilized questionnaires along with clinical assessments of post-secondary students by a professional odontologist.
(Ferreira da Silva et al., 2022) (10)	Brazil	Cross-sectional study with an observational and quantitative approach. Post-secondary students were interviewed and provided information about their participation in educational oral health actions, if they use private or public dental services, their perceptions of oral health, and their last visit.
(Benedito et al. 2020) (12)	Brazil	Descriptive study with a qualitative approach that utilized a questionnaire as well as written statements to collect information from post-secondary students.
(BenGhasheer & Saub, 2020) (23)	Malaysia	Descriptive, quantitative, cross-sectional study that measured acculturative stress, perceived stress, social support, oral health perceptions, and oral health-related quality of life.
(Oh et al., 2019) (16)	Korea	Cross-sectional study with self-administered questionnaires distributed to post-secondary students. Surveys included 4 general, 12 oral health-related, and 11 cultural adaption stress questions.
(Ohsato et al., 2018) (19)	Japan	Cross-sectional study that utilized student medical records.
(Mackert et al., 2017) (14)	U.S.	Cross-sectional online survey study. Survey investigated various patient factors such as perceptions of health insurance, barriers, and experiences.
(Jin et al., 2016) (17)	Korea	Cross-sectional study with self-administered questionnaires.
(Dewald, 2016) (15)	U.S.	Compiled data acquired from both ACHA-NCHA I and II (American College Health Association's National College Health Assessment).
(Park et al., 2014) (18)	Korea	Interview questionnaire was utilized to gather information on oral health knowledge and behaviours.
(Ogah, 2001) (13)	U.S.	Descriptive study utilizing a survey that post-secondary students had to mail-in.

Of the included studies, various designs were used (Table 1). In total, 13 studies used a cross-sectional design implementing surveys or interviews, while one additional study was a cross-sectional study that used surveys and objective measures (22). Only two studies did not distribute surveys for participants, one of which was a retrospective review and the other was a descriptive study that used a qualitative approach (12, 20). In total, four studies presented objective clinical measures (11, 19, 20, 22), whereas ten studies relied solely on self-reported information (10, 12–18, 21, 23). As well, eight studies exclusively provided data on international students (11–13, 15, 17, 18, 21, 23), while six studies compared domestic and international students in some capacity (10, 14, 16, 19, 20, 22).

In total, 13 studies had sample sizes below 1,000 participants (range: 40–826) (Table 2); of note, the studies with the largest and smallest sample sizes focused exclusively on international students. The remaining study was a 13-year longitudinal study conducted in the United States which included 60,453 participants from the American College Health Association's National College Health Assessment (ACHA-NCHA) (15).

**Table 2:** Included article objectives, sample sizes, population demographics and key results.

Author(s), date	Study objective	Sample size and population demographics	Key results
(Susanti et al., 2023) (21)	Determine the association of knowledge and attitude toward oral healthcare behavior of overseas university students	Nationality of International Students: Asian & non-Asian international students	- Overseas university students’ oral healthcare habits showed a positive correlation with their knowledge and attitude. - A negative correlation was found between their behavior and the incidence of oral health problems. - Students enrolled in health science programs demonstrated improved knowledge and attitudes toward oral health, while dental treatment coverage through insurance influenced their decisions to seek dental care.
		Sample Size: *n* = 311 international students	
		Age: Average age 27.5 years old	
		Gender: - Male (55.6%) - Female (44.4%)	
(Tsitaishvili & Jikia, 2023) (22)	The aim of this study was to assess the periodontal status of Caucasus International University (CIU) students of different nationalities, study the risk factors, and oral health optimization by planning and implementing preventive measures	Nationality of internationality students: Not specified	- Foreign students exhibited poorer gum health with higher bleeding on probing (BOP) degrees. - Lower rates of dental visits and tooth brushing frequency from foreign international students compared to their domestic counterparts. - 34.8% of Georgians and 66.4% of foreigners had gum bleeding.
		Sample Size: Total number of students (*n* = 820) *n* = 370 Georgian (Domestic) *n* = 450 foreigners (International)	
		Age: 18–35 years old	
		Gender: Domestic and international: - Male (*n* = 369) - Female (*n* = 451)	
(Abe et al., 2023) (20)	Compared the periodontal health status of international and domestic university students in Japan	Nationality of International students: Most from Asia (84.8%) - China *n* = 56 (83.6%) - South Korea with *n* = 4 (6.0%) - Singapore and Thailand both with *n* = 2 (3.0%) - Hong Kong, Taiwan, and Malaysia with *n* = 1 (1.5%)	International university students have poorer periodontal health than domestic students in Japan: - International students had a significantly higher percentage of BOP compared to domestic students and they exhibited more extensive calculus deposition. - However, there was no significant difference in probing pocket depth (PPD) between the two groups.
		Sample Size: Total number of students (*n* = 231) *n* = 79 international students *n* = 152 domestic students	
		Age: ≤25 and >25	
		Gender: Domestic Students (*n* = 152) - Male *n* = 118 - Female *n* = 34 International Students (*n* = 79) - Male *n* = 36 - Female *n* = 43	
(Ferreira da Silva et al., 2022) (10)	Aimed to characterize different socioeconomic and demographic contexts regarding the use and access of dental services by Brazilian and African students at a Brazilian university of international nature	Nationality of International students: Angola, Cape Verde, Guinea-Bissau, Mozambique, São Tome ´ and Príncipe, and East Timor	-Domestic students have better perception of their oral health, use of dental services, and frequent visit to dentist then international students.
		Sample Size: Toal number of students (*n* = 350)	
		Age: Under 25 years old	
		Gender: - Male (55.6%) - Female (44.4%)	
(Cruz et al., 2022) (11)	To evaluate the relation between the ingestion of cariogenic foods by African university students, and presence of dental caries, Candida spp., and salivary pH	Nationality of International students: African (Community of Portuguese language countries, CPLP) - Guinea-Bissau, Angola, Cape Verde, Mozambique and São Tomé and Príncipe	- Study showed that more than 80% of the international students consumed sugary foods due to the difficulties in managing academic activities or the reduced knowledge of the effects these foods have on oral health. - Despite ingesting cariogenic foods, even with a weekly frequency, students had low DMFT Index, adequate salivary pH and Candida was not present.
		Sample Size: *n* = 133 international students	
		Age: Average age 24.1 years old	
		Gender: - Male (63.91%) - Female (36.09%)	
(Benedito et al., 2020) (12)	Investigate the importance of oral health, and the frequency and the means used to perform oral cavity cleaning, and assess the knowledge and behavior of undergraduates towards oral diseases	Nationality of International students: Guinea-Bissau, Mozambique, São Tomé and Príncipe, Angola and Cape Verde	- International students value oral health for disease prevention, aesthetics, self-esteem, oral functionality, systemic health, food consumption, and overall physical health. - Daily oral hygiene was seen as essential, using a brush, toothpaste, and dental floss. - Students had insufficient understanding of oral pathologies and typically sought treatment only when a disease was already present.
		Sample Size: *n* = 40 international students	
		Age: Not specified	
		Gender: Not specified	
(BenGhasheer & Saub, 2020) (23)	Aimed to investigate the relationships between acculturative stress, perceived stress, social support, and subjective oral health outcomes among international graduate students in Malaysian public universities	Nationality of International students: Not specified	- Results indicated that acculturative stress, perceived stress, and social support are among the predictors of OHRQoL. - International students with high acculturative stress levels had a greater impact on OHRQoL. Psychosocial factors have a significant role in predicting and explaining subjective oral health outcomes.
		Sample Size: *n* = 312 International students	
		Age: Mean age 33.6 [standard deviation (SD) = 7.1]	
		Gender: - Female *n* = 111 (35.6%) - Male *n* = 201 (64.4%)	
(Oh et al., 2019) (16)	A cultural adaptation stress refers to such distress, confusion and negative behaviors that arise when one goes through the process of adapting to another culture This study examines the relationship between cultural adaption stress and oral health-related factors	Nationality of International students: Vietnamese	- Korean students (domestic) demonstrated higher oral health satisfaction and knowledge scores compared to Vietnamese international students. - Vietnamese students faced significant cultural adaptation stress, particularly related to language and social acceptance, which negatively impacted their oral health behaviors. - Those with better oral health habits, such as brushing three times daily, experienced significantly lower levels of cultural adaptation stress.
		Sample Size: Total number of students (*n* = 179) *n* = 80 Vietnamese (International) *n* = 99 Korean (Domestic)	
		Age: Not specified	
		Gender: International Students: - Male (52.5%) - Female (47.5%) Domestic Students: - Male (29.3%) - Female (70.7%)	
(Ohsato et al., 2018) (19)	The object of the present study was to clarify the current oral status of international university students	Nationality of International students: 1. Asian countries 88.4% (*n* = 122) - China (47.1%) - Korea (10.9%) 2. North America (5.8%) 3. Europe (4.3%) 4. Africa (1.5%)	- No significant difference was found in the history of dental treatment between international and non-international students. - International students had a higher rate of dental caries morbidity, DMFT and calculus deposition compared to non-international students. - Overall International students had poorer oral health status compared to their non-international counterparts.
		Sample Size: Total number of students (*n* = 554) *n* = 138 international *n* = 416 non-international students	
		Age: International students: - Average age 28 ± 3 years Non-international students: - Average age 25 ± 4 years Overall: - Mean age of 26 ± 4 years	
		Gender: - Male (74.5%) - Female (25.5%)	
(Mackert et al., 2017) (14)	Explore perceived barriers to using health insurance and identify discriminant factors between health insurance information seekers and non-seekers	Nationality of International students: Not specified	- Both domestic and international student sought health insurance information due to worry about negative consequences and perceived self-efficacy to process information in using health insurance. - Common barriers included cost and lack of understanding of how to use health insurance. For international students, language ability was an additional barrier.
		Sample Size: Domestic Students (*n* = 495) International Students (*n* = 120)	
		Age: From 17 to 39 years old (M = 21.09; SD = 3.40)	
		Gender: Domestic Students (*n* = 495) - Male *n* = 168 - Female *n* = 326 International Students (*n* = 120) - Male *n* = 66 - Female *n* = 54	
(Jin et al., 2016) (17)	This study aims to provide the basic data for ideal oral care behavior by identifying oral health behavior and oral health belief for Chinese students in Korea	Nationality of International students: Chinese	- Susceptibility and seriousness varied depending on the year of entry into Korea, which appears to show that, as students adapt better to university life in Korea with passing year, they feel differently about oral health belief. - Chinese international students feel inconvenient to use domestic medical institutions and had difficulty maintaining oral health since they are not obligated to acquire health insurance in Korea.
		Sample Size: *n* = 231 international students	
		Age: Not specified	
		Gender: - Male (50.7%) - Female (49.3%)	
(Dewald, 2016) (15)	To investigate the dental health seeking practices of US college students	Nationality of International students: Not specified	- 63% percent of international students have the annual exam and cleaning compared to 34.32% who did not. - International students are in need of being connected to dentists in the city where the university is located.
		Sample Size: *n* = 60,453 international students	
		Age: Not specified	
		Gender: Not specified	
(Park et al., 2014) (18)	To identify the oral health knowledge and perception of Chinese students’ studying in Korea who received the oral health education program	Nationality of International students: Chinese	Inconclusive results.
		Sample Size: *n* = 94 international students	
		Age: Not specified	
		Gender: - Male *n* = 56 - Female *n* = 38	
(Ogah, 2001) (13)	Examined possible changes in nutrition and dental practices of international students after migration to the United States (US)	Nationality of International students: From over 60 different countries	- All international students brush their teeth at least once a day, with many increasing to more than once daily after coming to the US. - Tooth flossing is not popular among international students, possibly due to a lack of awareness in many developing countries. - There was a significant decrease in dental check-ups among international students after arriving in the US, likely due to difficulties navigating the new environment and lack of transportation.
		Sample Size: *n* = 81 international students	
		Age: 85% over 21 years old	
		Gender: - Male (35%) - Female (65%)	

#### Participant characteristics

The age of participants from the included studies varied (mean age range: 17–39 years) (Table 2); five studies had a mean age of participants younger than 25 years (10, 11, 13, 14, 20), two studies had a participant mean age of 25–28 years (19, 21), and one study reported a participant mean age of 33.6 years (23). Eight studies had a higher proportion of male participants compared to female participants (10, 11, 14, 17–21, 23), Four studies had more female participants compared to male participants (13, 14, 16, 22), and two studies did not specify the gender distribution of included participants (Table 2) (12, 15). Lastly, eight studies did not specify the level of study of included international student participants (14–20, 22).

### Study findings

Information on key study findings is reported in Table 2.

#### Knowledge and attitudes towards oral health and oral health preventative behaviours

Overall, ten studies captured information on the knowledge and/or attitudes of international students regarding oral health and found varied results (10, 12–14, 16–19, 21, 22). Studies on knowledge and attitudes will be described separately.

In total, nine studies captured information on self-reported knowledge of international students regarding varied aspects of oral health (10, 12, 13, 16–19, 21, 22). In a study in Thailand (*n* = 826), over 70% of international student participants self-reported they were well-informed about dental caries (21). Fewer participants had awareness and understanding of gum disease, with only 60% of students having correct knowledge of gingivitis and how to prevent its disease progression (21). However, Park et al. (18), who studied Chinese international students in Korea (*n* = 94), reported that 71.3% of participants incorrectly answered dental decay is a preventable disease, and 72.3% incorrectly answered it is best to brush immediately after a meal. A study by Susanti et al. found that most international students thought that dental caries and gum bleeding were normal, with upwards of 90% of international students having this misunderstanding (21). The results from Park et al. (18) were similar, with 69.1% of Chinese international students incorrectly answering an assessment question on whether using dental floss helps prevent dental caries. A study conducted in Korea with Vietnamese international students on knowledge and attitudes also found that domestic (Korean) students scored 1.424 times higher in oral health satisfaction and 3.488 times higher in oral health knowledge, the latter being statistically significant, compared to international students (16).

#### Behaviours

In total, seven studies examined the self-reported oral health behaviours of international students (10, 13, 15, 16, 19, 21, 22). These self-reported behaviours are categorized into three main areas including flossing, brushing, and dental visits.

#### Flossing

Self-reported flossing behaviours of international students were described in three articles (13, 21, 22). A study conducted in the United States by Ogah examined changes in the self-reported flossing habits of international students before and after they arrived in their new country (13). They found non-significant improvements (*p* = 0.131) in flossing habits, with a slight decrease in the percentage of participants who never floss (from 39% to 32%) and a slight increase in the percentage of participants who floss less than once per day (from 31% to 37%) (13). A study carried out in Georgia by Tsitaishvili and Jikia found that self-reported flossing habits among international students were significantly lower compared to domestic students, with 8.6% of international students flossing regularly compared to 24.7% of domestic (Georgian) students (*p* < 0.01) (22).

#### Brushing

Self-reported brushing habits were reported in three studies. A Korean study by Oh et al. (16) found that 78.8% of Vietnamese International students brush their teeth 1–2 times daily and 13.8% do not brush at all (16). A 1999 survey study of international students by Ogah conducted in the United States found the percentage of international students brushing more than once per day increased from 76% to 84%, while those brushing once per day decreased from 24% to 17% (13). These changes were statistically significant (*p* = 0.031). Like flossing, the article by Tsitaishvili and Jikia conducted in Georgia found brushing habits among international students were lower compared to domestic students. These authors found 82.8% of international studies brush their teeth daily vs. 95.3% of domestic (Georgian) students (*p* < 0.01) (22).

#### Dental visits

Frequency of dental visits among international students was reported in four articles (10, 13, 19, 22). The frequency of dental visits among international students was generally found to be lower compared to domestic students. The study by Tsitaishvili and Jikia from Georgia found only 31.8% of international students visited a dentist once a year and just 2.7% twice yearly (*p* < 0.01) (22). In addition, the study by Ogah found a significant increase in the percentage of international students studying in the USA who never visited a dentist (increase from 20% to 52%) and significant decrease in the percentage of international students reporting yearly visits (decrease from 38% to 18%) (*p* < 0.001) (13).

Of the four studies that examined frequency of dental visits, two reported on differences in dental visit frequency between international and domestic students. Ohsato et al. (19) found that international students were less likely to seek routine dental checkups (30.1%) compared to domestic students (48.6%) (19). Lastly, a study conducted in Brazil by Ferreira da Silva et al. (10) reported that 53.96% of international students use dental services compared to 100% of domestic (Brazilian) students (*p* < 0.05), and only 52.29% of international students visited a dentist in the last year in contrast to 70.95% of domestic (Brazilian) students (*p* < 0.05).

#### Dietary intake

In total, three included articles investigated the dietary intake of international students (11, 13, 21). A recent study conducted in Brazil by Cruz et al. (11) revealed that over 90% of international students reported consuming sweetened cookies and desserts. The frequency varied, with 29.23% of students consuming these foods once a week. Additionally, the study found 82.0% of international students consumed sugary foods, and nearly 90% ingested powdered juices (11). No statistically significant differences in DMFT were found between groups categorized based on various dietary intake measures.

Two included studies that captured information on dietary intake also compared the students' diets before and during their stay in the foreign country (13, 21). The work by Ogah highlights the transitional challenges faced by international students, with international students more frequently consuming sugar-containing foods, high-fat foods, fast food, and fewer fruits and vegetables after arriving in the United States compared to before their arrival (13). More specifically, the percentage of students who ate no fast food decreased from 43% to 17% (*p* < 0.01), the percentage of those who consumed fast food 3–5 times per month doubled from 14% to 28%, and the percentage of international students consuming fast food 6–10 times per month nearly tripled from 4% to 15% (13). Additionally, a study from Thailand conducted on international students studying at Chulalongkorn University found that 40% of students consumed more sugar-containing foods and drinks after moving abroad, and 60% reported consumption occurring at high risk times for dental caries (e.g., before bed) (21).

#### Acculturation and stress

In total, two studies (16, 23) investigated associations between stress and different measures of oral health. A study by BenGhasheer and Saub investigated the associations between acculturative stress (i.e., process of adapting to a new culture) on the oral health-related quality of life (OHRQoL) of international students' studying in Malaysian public universities (23). They found a significant association between acculturative stress and OHRQoL, where higher levels of acculturative stress were associated with poorer OHRQoL (23). They also reported that acculturative stress affected students OHRQoL both directly and indirectly through increased perceived stress.

A study by Oh et al. (16) on Vietnamese international students studying in Korea further illustrates the specific stresses related to cultural adaptation (e.g., pressure to learn the Korean language) are identified as the most stressful factors. Students who adhered to regular oral hygiene practices, like brushing their teeth three times daily, reported significantly lower levels of cultural adaptation stress compared to those who did not.

Additionally, social support emerged as a crucial factor in mitigating the effects of acculturative stress on OHRQoL (23). BenGhasheer and Saub's research described earlier found social support mediated the relationship between perceived stress and OHRQoL, with higher levels of social support predicting lower levels of both perceived stress and acculturative stress (23).

#### Oral health status

In total, four studies described the oral health status of international students (19–22). The trend consistently observed throughout the reviewed literature was that international students experienced issues such as gum bleeding, high rates of decayed, missing, and filled teeth (DMFT index), and calculus deposition (19, 20, 22).

Overall, two studies investigated objective differences in oral health status in international students compared to domestic students (20, 22). International students tended to exhibit poorer oral health compared to their domestic student counterparts (20, 22). Differences in gum bleeding between domestic and international students were noted in two articles from Georgia and Japan (20, 22). Tsitaishvili and Jikia found the proportion of students with bleeding on probing (BOP) was greater among international students compared to domestic (Georgian) students (66.4% of foreign students vs. 34.8% of domestic Georgian students (22). In Japan, Abe et al. reported 49.4% of international students experienced BOP vs. 34.2% of domestic students (*p* < 0.05) (20). In addition, two studies examined differences in calculus deposition between international students and domestic students; they both found international students had more extensive calculus deposition compared to domestic students (19, 20). In addition, one of the two studies found that international students had a higher proportion of missing teeth and dental caries compared to domestic students. Furthermore, the proportion of international students with poor dental hygiene was 59.0%, while for non-international students, it was 33.8% (*p* < 0.05) (19). Ohsato et al. (19) also found that international students had a higher mean DMFT value vs. domestic students (5.0 vs. 4.0).

#### Dental insurance and costs

In total, three studies described a relationship between dental visits and insurance coverage (14, 21, 22). According to a study on predictors of periodontal diseases among university students, financial considerations significantly influenced the frequency and type of dental care sought by students (22). The study revealed most students only sought dental care in response to pain or discomfort, rather than for preventive measures (22). A study in Thailand further identified cost as another barrier, where 11.6% of international students expressed hesitancy in visiting the dentist because of cost of treatment (21). In addition, a comparative analysis of domestic and international students' health insurance information-seeking behaviour revealed international students often face barriers in understanding and utilizing health insurance (14). This confusion is exacerbated by the complex nature of insurance systems in their host countries and the additional challenge of navigating these systems in a non-native language (14).

## Discussion

This scoping review aims to study existing literature on oral health knowledge, attitudes, behaviours, barriers, and status among international post-secondary students. While some reviews have been previously completed on health issues among international students (e.g., mental health, diet, sexual and reproductive health, stress) (24–30), to our knowledge, a review on oral health does not exist.

The literature reviewed was predominantly cross-sectional and offered valuable insights into the attitudes and behaviours of international students. However, this point-in-time approach limits the ability to assess the long-term outcomes for these individuals. Given that international students often reside in their host countries for extended periods, understanding how their oral health behaviours and status evolves over time—whether it improves or deteriorates—would be highly beneficial. Only two articles took into account the duration of international students' stays in foreign countries (17, 21); of note, these studies were not consistent in their metrics, with one providing duration of stay in months and years while the other article reported changes before or after a specific year (2010) (17, 21). Other gaps found in the included articles were the lack of reporting of education levels, marital status, and more comprehensive insurance coverage information which would help to better determine the accessibility to care.

Moreover, very few studies employed objective measures (e.g., DMFT, objective periodontal measures) to assess oral health status; this may reduce the reliability of the findings, as these measures can provide a more accurate and unbiased reflection of participants' true oral health status. Additionally, since most of the studies were cross-sectional and relied primarily on self-reported survey data, they are vulnerable to several biases. Recall bias might have occurred if participants were unable to accurately remember their past behaviours, leading to an overestimation or underestimation of activities (e.g., frequency of brushing, flossing teeth, dietary behaviours) (31). Social desirability bias could also be a concern, as participants may provide answers they believe are socially acceptable or expected, rather than report true behaviours (31).

The reviewed literature includes students studying in various host countries across the globe. However, they provide limited details on whether their previous or current dental care was received in their home countries or their place of study; this missing information may result in overlooking key contextual factors that can impact behaviours and/or oral health outcomes. Access to dental care, service quality, cultural attitudes, and dental insurance availability can vary significantly between countries. These factors are important considerations for future research to understand if students are unable to access dental care in their country of study and/or if they return to their home country to receive care.

Moreover, none of the included studies captured information on water fluoridation. It is noteworthy that both the home and host countries of many international students could offer fluoridated water to portions of their populations which is relevant to oral health status (32). However, it would have been insightful to determine whether the students themselves had access to fluoridated water. To facilitate a more robust comparison between international and domestic students, examining the accessibility of water fluoridation within these groups would be instrumental in elucidating disparities in oral health outcomes.

Although some of the included articles provided information about dietary habits relevant to oral health, the information that was captured was limited and primarily focused on a few foods (e.g., sugary drinks, fast foods). Having this data would be important as oral health status is highly linked to numerous dietary factors that goes beyond sugar and sugary beverages, including intake of calcium, vitamin D, fluoridated water, and overall diet quality (e.g., vegetables, fruit, grain products, protein foods); future studies may want to consider capturing more comprehensive information. In addition, information about knowledge, attitudes, and perspectives regarding diet and oral health was not captured in these included articles. Lastly, no included studies captured information on food insecurity which we know has a higher prevalence in international students compared to domestic students (24). This lack of information is important as food insecurity has been previously linked to poorer oral health and unmet dental needs (33–35). Since dietary habits and food insecurity are important determinants of oral health, prioritizing the collection of this information in future studies is critical.

Furthermore, given these initial findings identifying that international students encounter challenges in maintaining their oral health status, it will also be essential to consider future targeted interventions that could improve experiences and outcomes in oral health for this population. Future studies can also provide the information that is needed to effectively inform initiatives at educational institutions and government bodies (policy support) to optimize oral health and foster overall health and wellness of international students. Such initiatives should be designed to empower the students to navigate the challenges of adaptation into the new environment and become well-rounded, resilient and healthy members of their new communities.

## Strengths and limitations

This review had several strengths. We employed a robust methodology (Arksey and O'Malley) and searched six major databases (assisted by librarian) to enable a thorough and exhaustive exploration of the available literature. As well, articles were searched using other channels (e.g., reference lists of included articles, citations). Moreover, we also carefully screened the titles and abstracts using software with multiple reviewers.

Our review had some limitations. We did not consider grey literature (non-peer-reviewed materials such as reports, and conference papers), and we restricted our analysis to English-language articles, thereby excluding some research that might have provided a broader, more diverse perspective. We also made the methodological choice to not undertake a formal quality appraisal of the included studies, which may impact the robustness and reliability of our conclusions.

## Conclusion

International students may face significant challenges in managing and optimizing their oral health compared to domestic students. These differences may stem from a variety of factors ranging from cultural differences in upbringing to stress levels, dietary intake, food insecurity, to awareness and accessibility of supports in their host countries. Post-secondary institutions may want to consider focusing on supporting and empowering international students to access oral health care. Some ideas could include offering informative workshops and campaigns (e.g., oral health awareness campaigns), establishing mentorship programs with domestic students, and more advertising regarding oral health supports and services available on and off campus. These approaches could facilitate smoother integration into the new environment and better oral health status for international students.

More community engaged research is needed, including qualitative research, to understand the perspectives of international students regarding their oral health status, knowledge, attitudes, behaviours, needs, and aspirations; this information is needed to determine how to design future impactful interventions in this area. As well, because the oral health care landscape varies between countries and even regions within a country, it is important that research in this area is conducted in a variety of settings to better understand how to support international students to optimize their oral health status. In addition, more studies are needed to capture information on objective measures, and more comprehensive information about behaviours that impact oral health status (e.g., brushing, flossing, diet, food insecurity) to create a more holistic picture of oral health and its linkages to overall health in this vulnerable population.
