# Supplementary material for: Oral health knowledge, attitudes, behaviours and status among international post-secondary students: a scoping review
Source: Front Oral Health. 2025 Mar 21;6:1555165. doi: 10.3389/froh.2025.1555165 (PMC11968716; doi:10.3389/froh.2025.1555165)
Supplement: Supplementary file 1 [file Table1.docx]

**Supplementary Material 1: Search Strategy**

| **Database** | **Search Terms** |
| --- | --- |
| Medline | ***Oral health related terms:***  Keywords: “oral health*”.ti,ab,kw OR periodont*.ti,ab,kw OR oral hygiene.ti,ab,kw OR dent*.ti,ab,kw OR tooth.ti,ab,kw OR teeth.ti,ab,kw OR gingiv*.ti,ab,kw  MeSH terms:  oral health/ OR  exp periodontal diseases/ OR  exp tooth diseases/ OR  exp dentition/ OR  dental health services/ OR  dental care/ OR  oral hygiene/ OR  exp dentistry/ OR  exp public health dentistry/ OR  insurance, dental/  ***Student related terms:***  Keywords: overseas adj2 student*.ti,ab,kw OR international adj2 student*.ti,ab,kw OR foreign-born adj2 student*.ti,ab,kw OR exchange adj2 student*.ti,ab,kw OR foreign adj2 student*.ti,ab,kw OR study abroad student*.ti,ab,kw OR international adj2 scholar*.ti,ab,kw OR international adj2 learner*.ti,ab,kw OR international adj2 trainee*.ti,ab,kw OR abroad adj2 student*.ti,ab,kw |
| Embase | ***Oral health related terms:***  Keywords: “oral health*”.ti,ab,kw OR periodont*.ti,ab,kw OR “oral hygiene”.ti,ab,kw OR dent*.ti,ab,kw OR tooth.ti,ab,kw OR teeth.ti,ab,kw OR gingiv*.ti,ab,kw  Emtree terms:  exp dental health/ OR  exp tooth disease/ OR  exp dentition/ OR exp dental procedure/ OR exp dental facility/ OR exp dentistry/ OR exp dental health education/ OR exp dental insurance/  ***Student related terms:***  Keywords: overseas adj2 student*.ti,ab,kw OR international adj2 student*.ti,ab,kw OR foreign-born adj2 student*.ti,ab,kw OR exchange adj2 student*.ti,ab,kw OR foreign adj2 student*.ti,ab,kw OR study abroad student*.ti,ab,kw OR international adj2 scholar*.ti,ab,kw OR international adj2 learner*.ti,ab,kw OR international adj2 trainee*.ti,ab,kw OR abroad adj2 student*.ti,ab,kw  Emtree terms:  exp foreign student/ |
| Dental and Oral Science Sources | ***Student related terms:***  Keywords:  overseas W2 student* OR international W2 student* OR foreign-born W2 student* OR exchange W2 student* OR foreign W2 student* OR study abroad student* OR international W2 scholar* OR international W2 learner* OR international W2 trainee* OR abroad W2 student* |
| CINAHL | ***Oral health related terms:***  Keywords: **“**oral health*” OR periodont* OR “oral hygiene” OR dent* OR teeth OR gingiv*  Controlled terms:  MH "Oral Health" OR MH "Tooth Diseases+" OR MM "Insurance, Dental" OR MH "Dentistry+" OR MH "Dental Health Services+" OR MM "Dental Health Education" OR MH "Oral Hygiene+"  ***Student related terms:***  Keywords:  overseas W2 student* OR international W2 student* OR foreign-born W2 student* OR exchange W2 student* OR foreign W2 student* OR study abroad student* OR international W2 scholar* OR international W2 learner* OR international W2 trainee* OR abroad W2 student*  Controlled terms:  MH "Students, Foreign" |
| Web of Science | ***Oral health related terms:*** ALL=("oral health*" OR dent* OR gingiv* OR periodont* OR teeth* OR tooth* OR "mouth disease*") ***Student related terms:*** ALL=("international student*" OR "foreign student*" OR "overseas student*" OR "exchange student*") |
| Scopus | ***Oral health related terms:*** Keyword:  "oral health" ***Student related terms:*** Keyword:  "international students" |
